# Hormone residues are not detected in zero water exchange biofloc system during tilapia masculinization even in higher feed input

**DOI:** 10.1590/1984-3143-AR2025-0106

**Published:** 2026-02-16

**Authors:** Dara Cristina Pires, Érika Ramos de Alvarenga, Franklin Fernando Batista da Costa, Kelly Moura Keller, José Fernandes Bezerra, Mariana Parrini Ferreira, Gabriela Lago Biscoto, Isabela Lopes Samary, Karen Beatriz Guerra Lima, Vinícius Monteiro Bezerra, Caroline Lopes de Melo, Lee Deyver Carvalho Pena Mansur, Williane Ferreira Menezes, José Fernando Paz Ramírez, Luiza Fujii Almeida, Daiana dos Reis Pelegrine, Marcelo Rezende Luz, Eduardo Maldonado Turra

**Affiliations:** 1 Escola de Veterinária, Universidade Federal de Minas Gerais, Belo Horizonte, MG, Brasil; 2 Empresa de Pesquisa em Agropecuária de Minas Gerais, Felixlândia, MG, Brasil; 3 Instituto de Ciências Biológicas, Universidade Federal de Minas Gerais, Belo Horizonte, MG, Brasil

**Keywords:** methyltestosterone residues, tilapia sexual inversion, zero water exchange, stocking density, biofloc

## Abstract

How intensive the masculinization period of Nile tilapia can be in biofloc technology (BFT) based on higher stocking densities and a zero-water exchange protocol to mitigate the environmental impact has not been studied yet and needs investigation. Thus, our objective was to determine the optimal stocking density for reduction of hormonal effluent in BFT, better growth and masculinization rate of Nile tilapia larvae, and lower total variable cost. Five stocking densities (1.5, 3, 4.5, 6, and 7.5 larvae ∙ L^-1^) with four replicates were tested. Tilapias were fed with 60 mg of 17α-methyltestosterone (MT) ∙ Kg^-1^ of feed for 28 days, and with hormone-free feed until the common selling body weight of 1g. Water quality variables showed no significant differences between treatments, except for total settleable solids, pH, and final total organic carbon, that had a negative, positive and quadratic linear pattern with the increasing of stocking density, respectively. Growth performance variables such as final body weight, specific growth rate, survival and individual feed intake (and consequently hormone input) were superior at the lower stocking density. Masculinization rate (98.87%) did not differ among treatments, but total variable cost increased to produce 1g fingerlings in higher stocking densities. Hormone residue was not detected in BFT water of any treatment 12 hours after the last feeding so, in conclusion, even being the highest hormone input in the system, 1.5 larvae ∙ L^-1^ is the best level of stocking density studied for tilapia masculinization in a zero-water exchange BFT.

## Introduction

Several strategies are employed for reproductive control of Nile tilapia, one of the most common approaches being the production of monosexual populations through the masculinization of individuals with the use of 17α-methyltestosterone (MT) (Baroiller and D’Cotta, 2018; El-Sayed, 2019; Costa e Silva et al., 2022; Ramírez et al., 2024). This technique is widely used due to its affordable cost, availability of hormone, and high efficacy in inducing males (Baroiller and D’Cotta, 2001). The tilapia masculinization protocol commonly used was reviewed by Baroiller and D’Cotta (2018), who recommend the use of 50-60 mg of MT∙ kg^-1^ of feed, during 28-30 days post-hatching, with stocking densities of 3-10 larvae∙ L^-1^. Sexual differentiation in this species occurs between 5 and 21 days post-hatching, a period that is critical for the influence of abiotic factors (environmental or hormonal) on gonadal development (De Alba et al., 2024). However, since the collected larvae used for masculinization may vary in age, masculinization protocols are typically designed to cover and extend beyond this period (lasting 28 to 30 days) to ensure process efficiency (Baroiller and D’Cotta, 2018).

Traditionally, tilapia masculinization is performed in earthen ponds—either within hapas or not—with partial water exchanges. This system is widely adopted due to its operational simplicity and low implementation cost (Popma and Green, 1990; Contreras‐Sánchez et al., 2001; Silva et al., 2023). However, feed leftovers containing unmetabolized hormones may remain in the culture pond or tanks and, if released as effluent, have the potential to contaminate surrounding discharge points (Homklin et al., 2012). Furthermore, MT has endocrine disrupting properties that may interfere with the normal reproductive functions of other organisms, including humans (Liu et al., 2023). Thus, it is essential to develop alternative production systems that reduce environmental impacts and allow for a more environmentally sustainable tilapia masculinization process.

Biofloc technology (BFT), a closed aquaculture system, has emerged as a promising production alternative by allowing reduced water and land use, production at higher stocking densities (compared to ponds), minimal water exchange, and recycling of nutrients present in the water, resulting in lower pollutant emissions (Avnimelech, 2009; Crab et al., 2012; Ahmad et al., 2017; Alvarenga et al., 2017). Furthermore, recent studies conducted on the biodegradation of MT in sediments from tilapia masculinization tanks (Homklin et al., 2011; Homklin et al., 2012) suggest that bacterial conditioning may be an alternative to promote the natural degradation of this hormone and its residues. In this sense, the biofloc system, by harboring diverse bacterial communities, has the potential to optimize the MT degradation process.

BFT has been investigated as an alternative approach for tilapia masculinization on a laboratory scale (Costa e Silva et al., 2022; Costa et al., 2024; Ramírez et al., 2024). Its use in commercial operations has been reported only after the masculinization phase, in production units of 100 L (for tests) or larger (García-Rios et al., 2019). In such cases, the system is applied in tanks containing bioflocs, with minimal water exchange. Despite significant advances in the development of tilapia masculinization in BFT, several challenges remain, including the determination of optimal stocking density and the understanding of hormone biodegradation within this system (Ramírez et al., 2024), which could ultimately make it more attractive to commercial producers. Determining the optimal stocking density is generally associated with achieving the highest production efficiency per unit of area or volume, which involves maximizing body weight gain and minimizing feed conversion ratio within a relatively short cultivation period, while ensuring a commercially viable harvest weight (Schimittou, 1997), leading to a higher economic return on investments in infrastructure and equipment (Hengsawat et al., 1997). However, in the masculinization phase, the high proportion of males and hormone input and residues are very important indicators and the number of larvae per liter could interfere with these variables. To our knowledge, there are no published studies on the effect of stocking density during masculinization of Nile tilapia (*Oreochromis niloticus*) on hormone residues, performance indicators, survival, masculinization rate, water quality and production costs in BFT. In fact, there are not many studies in the literature that address the stocking density of tilapia larvae in the masculinization phase in any system, even in hapas installed in ponds or in the water recirculation system (RAS) (Tachibana et al., 2009; Sanches and Hayashi, 1999; Vera Cruz and Mair, 1994). Several of these studies were carried out in this phase of tilapia larviculture, with larvae with an initial weight of 0.01 g, but without the animals being subjected to masculinization treatment through hormones in the diet. Under these conditions, studies have already been carried out in RAS (Macintosh and Silva, 1984; Dambo and Rana, 1993; El-Sayed, 2002) and in a system with 80% water changes twice a day and in salinized water (Luz et al., 2012). More recently, stocking density was evaluated in BFT, but with tilapia with an average initial body weight between 3.2 and 4.79 g (Aliabad et al., 2022; Pai et al., 2024), that is, after the masculinization phase.

Increasing the stocking density in this system can lead to the accumulation of solids and nitrate in the water (Manduca et al., 2020), which is why a minimum water change of 5% per day is recommended (Manduca et al., 2021). However, during the larviculture phase, due to the reduced biomass of animals in the system, performing minimum water changes may not be necessary. Furthermore, considering the presence of hormonal residues that need to be safely disposed of in the environment, not performing water changes allows this residue to remain confined for later treatment. In studies carried out in this phase without water exchange, different aspects were analyzed, including feeding frequency and MT concentration in the diet (Costa e Silva et al., 2022), the use of alcohol and oil as vehicles in masculinization (Costa et al., 2024) and the reduction of MT concentration in the diet of Nile tilapia larvae in biofloc systems (Ramírez et al., 2024). All these studies achieved masculinization rates higher than 97% with a stocking density of 2 larvae ⋅ L^−1^.

Therefore, defining an ideal stocking density for the masculinization phase in an efficient and sustainable system requires a comprehensive analysis that considers growth performance, water quality and expenditure, production costs, masculinization rates, and hormonal residues. Thus, the present study aims to determine the optimal stocking density of Nile tilapia (*Oreochromis niloticus*) larvae reared during the masculinization phase in BFT with zero water exchange, based on these aspects.

## Methods

### Animals and experimental design

The experiment was conducted in a greenhouse at the Aquaculture Laboratory of Veterinary School of the Federal University of Minas Gerais (Laboratório de Aquacultura - LAQUA, Escola de Veterinária / Universidade Federal de Minas Gerais - UFMG), Brazil. All procedures were previously approved by the Ethics Committee on Animal Use of UFMG under protocol number 263/2023.

Four thousand e five hundred Nile tilapia larvae (4,500) at the same stage of development (right after yolk sac absorption) were randomly collected from the larvae production of 6 females and distributed into 20 polyethylene tanks with an effective volume of 50 L, selected to provide an appropriate balance between experimental control, repeatability, and representativeness of real farming testing conditions (García-Rios et al., 2019). Five densities were used: 1.5, 3, 4.5, 6, and 7.5 larvae ∙ L^-1^, with four replicates each. A control group fed with a hormone-free diet and based on a stocking density of 2.0 larvae ∙ L^-1^ (according to Costa e Silva et al., 2022; Costa et al., 2024; Ramírez et al., 2024) was also raised on four tanks of the same useful volume. The control group was used as a reference of the proportion of males in larvae that were not fed with masculinizing hormone in the diet.

During the first 28 days of post yolk sac absorption, the Nile tilapia larvae were fed with a commercial ration (Propescado-Nutriave Foods) containing 55% crude protein, 12% moisture, 10% total lipids and 15% ashes, enriched with MT, except in control group. The concentration of 60 mg of MT ∙ Kg^-1^ of feed was chosen considering the study of Costa e Silva et al. (2022) of Nile tilapia in BFT. After 28 days, the fingerlings were fed a hormone-free diet until reaching a body weight of approximately 1g.

The hormone was weighed in a precision balance of 0.001g (©Marte Científica, Brazil) and dissolved in 200 mL of 99.5% P.A ethyl alcohol, then sprinkled to the larvae feed during a mixing process. The feed was then stocked in a dark room for alcohol evaporation and hormone fixation, and after 24 h the fish feeds were stored in black containers. The containers were stored in a freezer under − 20 ºC, to keep hormone levels throughout the trial according to Barry et al. (2007).

The larvae were fed five times a day and the feeding rate applied was 30% of the larvae biomass for the first week, 25% for the second, 20% for the third, and 15% for the fourth week, as described by Costa e Silva et al. (2022). For the first week, the biomass of each tank used to estimate the amount of feed to be offered was based on an expected growth of the larvae 3 times its initial body weight. For the second week, the biomass of each tank was estimated by the weighing of 20 larvae at the end of the first week and an expected proportional growth that each tank has had. For the third and fourth weeks, the procedures were the same, however the number of fish in each tank used for the biomass estimation was adjusted after a count made at the end of the second week.

### Water quality

The 24 tanks were initially filled with 50 L of water containing bioflocs previously developed in our laboratory, presenting an initial settleable solids level of 8 ml ∙ L^-1^ and total ammonia nitrogen (TAN) and nitrite (NO_2_^−^) concentrations of 0.01 mg ∙ L^-1^ and 0.275 mg ∙ L^-1^, respectively. Heaters with thermostats were used to maintain minimum water temperature at 28ºC. Dissolved oxygen (DO) was measured two times per week using the AT 155 oximeter (Alfakit®, Florianópolis, Santa Catarina, Brazil). Temperature, salinity, and pH were also measured two times per week using a multiparameter probe (Hanna®, Barueri, São Paulo, Brazil). TAN and NO_2_^-^ were analyzed two times per week using spectrophotometry (Biochrom Libra S22), according to UNESCO (1983) and Bendschneider and Robinson (1952), respectively. To determine the un-ionized ammonia (NH_3_, mg ∙ L^-1^), the formula NH_3_ = [NH_3_ + NH_4_^+^]/[1 + 10 (pKa-pH)] was used, where pKa = 0.09018 + 2729.92/(273 + water temperature (°C)) (Liu et al., 2008). Settleable solids (SS) and alkalinity were measured once a week through the Imhoff cone (Avnimelech, 2009), and protocol adapted from APHA (1998), respectively. Total suspended solids (TSS), nitrate, phosphate, and carbon were measured at the beginning and end of the experiment using the method described by Strickland and Parsons (1972), Monteiro et al. (2003), Murphy and Riley (1962) and by a Shimadzu TOC analyzer (TOC-L), respectively.

During the entire experimental period, there was no water renewal or solids removal from the tanks, with the volume of water lost through evaporation being supplemented, daily, with clear water from an artesian well. When TAN exceeded 0.5 mg ∙ L^-1^, cane sugar (estimated to be 50% carbon) was added to the experimental unit to maintain the proportion of 6:1 (C/N), according to Ebeling et al. (2006). Also, sodium chloride was added until a 1 g ∙ L^-1^ concentration was reached to reduce nitrite toxicity in all the tanks (adapted from Yanbo et al., 2006). The alkalinity was adjusted using calcitic limestone.

### Growth performance and sexing

Growth performance was evaluated based on the initial body weight (IW), final body weight (FW), final biomass (FB), final productivity (FSD), daily weight gain (WG), specific growth rate (SGR), total feed intake (TFI), average individual feed intake (FI) final length (FL) and survival (S). The variables were assessed at the end of the 28th day of the experiment, as follows: initial body weight (g), mean of the mass (g) of 20 randomly picked post yolk sac absorption larvae of each tank that was dried in a paper towel and weighed together for the initial body weight determination; final body weight (g), mean of the mass (g) of 30 larvae randomly picked per experimental unit; final biomass (g), final mean weight × final quantity of animals per tank; final productivity (kg ∙ m^-3^), FB / useful tank volume (0.05 m^3^); daily weight gain (mg ∙ day^-1^), [(FW – IW) / t × (1000)], where t is the duration of the trial (28 days); specific growth rate (% ∙ day^-1^), 100 × (log (FW) – log (IW)) / t; total feed intake (g), total feed offered; individual feed intake (FI), total feed offered / final quantity of fingerlings; survival (S), 100 × (final quantity of animals per tank / initial quantity of animals per tank).

After the 28 days of masculinization period, the fingerlings from each tank were raised until they reached a mean body weight close to 1g (ranged from 6 to 23 days between the experimental units), a very common selling weight on the Brazilian market (Oliveira et al., 2007). This was considered a second larviculture phase, where all stocking densities were adjusted to 1.5 fingerlings ∙ L^-1^ to evaluate the effect of stocking densities of the first phase (masculinization period) on the time each experimental unit would take and how much feed would be spent to reach the selling body weight (1g).

When the experimental unit reached the mean body weight target (1g), 30 fingerlings were individually weighed on a precision balance (©Marte Científica, Brazil), and 50 were randomly chosen for microscopic analyses of gonads and sex determination. These fish were stored in Bouin liquid for 24 h and then transferred to 70% alcohol. Acetocarmine squash technique described by Guerrero and Shelton (1974), validated by Wassermann and Afonso (2002) and reviewed by Makino et al. (2008) was used to identify males, females, undifferentiated and intersex fish. Fish were classified as intersex when they presented both spermatogonial cysts and oocytes. This method allows for the clear observation of gonadal structures with high accuracy. In addition, it enables the evaluation of a larger number of samples in less time and at lower cost, when compared to histological analysis, thereby reducing statistical error and increasing the representativeness of the results.

### Analysis of MT

The evaluation of the introduction of MT into the system was conducted considering the concentration of MT and the amount of feed offered in each treatment. The formula used was: MT input (µg ∙ mL^-1^) = feed offered (Kg) × 60,000 µg ∙ Kg^-1^ × 50,000 mL^-1^.

Water samples containing bioflocs and suspended fecal matter (unfiltered) were collected from each tank 12 hours after the last feeding with hormone-enriched feed and over the subsequent nine days.

Acetonitrile and methanol were of HPLC grade and obtained from Sigma Aldrich (St. Louis, MO, USA). Water was purified in-house with a Milli-Q water purification system (18.2 Ω, Millipore Co., MA, USA). All other chemicals were higher than analytical grade and obtained from commercial sources. The methyltestosterone (MT) standard was acquired from Active Pharmaceutica Ltda (Palhoça, SC, Brazil) with a purity of ≥ 99%. The standard solution was prepared in methanol at a concentration of 1,000 μg ⋅ mL^-1^. Working solutions were prepared through appropriate dilutions of stock solutions. All solutions were stored at -20°C for subsequent use.

Blank biofloc water samples were spiked with different concentrations of MT and a standard curve was fitted for the analytical method validation procedures. For extraction, equal volumes of methanol and biofloc water were added to test tubes and vortexed for 1 minute. The samples were then centrifuged at 6,000 rpm (rotor with a radius of 6.5 cm) for 10 minutes to allow sediment precipitation, followed by extraction and clean-up using solid-phase extraction (SPE) cartridges. The SPE cartridges were conditioned by passing 5.0 mL of acetonitrile through them, followed by 5.0 mL of Milli-Q water. All samples were extracted by passing them through the SPE cartridges at a flow rate of 2.0 mL/min. A washing step with Milli-Q water was performed before elution with 5.0 mL of HPLC-grade ethanol. The eluate was evaporated to dryness at 37-40 °C, and the residue was re-dissolved in 1,000 μL of mobile phase. The resulting extracts were used for chromatographic analysis.

Appropriate amounts of standard solution/working solution were added to blank matrix extracts to prepare the calibration standard series in a matrix of 50, 100 and 200 µg ⋅ L^-1^.

High-performance liquid chromatography (HPLC) analyses were performed using a JASCO LC-2000 Plus HPLC System. Chromatographic separation was performed using a C18 column in reverse phase mode. The mobile phase used was acetonitrile:methanol:water (50:10:40 v/v/v) in isocratic mode. The injected volume was 100 μL, the flow was 1.0 mL per minute, and the column temperature was set to 25 °C. MT was detected at 245 nm using a UV detector. ChromNAV 2.0 software was used for data acquisition and processing. Each sample was analyzed in triplicate.

Selectivity was evaluated by comparing chromatograms of blank samples with those spiked with MT. Matrix effect was assessed by studying the linearity of calibration curves in solvent solution and in matrix. The coefficient of correlation (r) and coefficient of determination (R^2^) were calculated. The limit of detection (LOD) and limit of quantification (LOQ) were determined. The limit of detection (LOD) represents the lowest concentration that produces a signal distinguishable from background noise and was calculated using a signal-to-noise ratio (S/N) of 3:1. The limit of quantification (LOQ) was determined as the lowest concentration that could be reliably quantified, calculated based on a S/N of 10:1. These values were derived from the analysis of three replicate samples. Method precision was evaluated through intra-laboratory repeatability and reproducibility studies. Accuracy was assessed by the recovery method with intentional addition.

### Cost evaluation

From current Brazilian market data, estimates of costs per kilogram of feed, cane sugar, limestone, and common salt were US$ 1.60; US$ 0.80; US$ 0.20 and US$ 0.15, respectively. The cost of the MT was US$ 6.40 ∙ g^-1^ and for each kWh it was US$ 0.15. The estimated cost of acquisition of the initial larvae was US$ 6.00 ∙ 1,000 ind.^-1^. All costs of each experimental unit were estimated for each live fingerling at the end of the 28-day masculinization period, and these results were used to estimate the costs of a thousand 28-day live fingerlings. The same procedure was used to estimate the costs of the production of a thousand live fingerlings of 1 gram of body weight, at the end of the second phase. However, as cane sugar, limestone and common salt presented less or no expenditure in both phases, they were not considered for the second one. The sum of all considered variable costs was denominated total variable cost.

The amount of energy used to ensure the oxygen needed for each tank in BFT was estimated by the sum of the energy needed to provide oxygen for bacteria and fish from each tank to the whole experiment period, according to Boyd and Tucker (1998), Avnimelech (2009) and Timmons and Ebeling (2010). The average amount of oxygen needed by the bacteria and the fish in the tank was one gram of oxygen per one gram of feed offered. According to Boyd and Tucker (1998), from the result of total grams of oxygen (g O_2_ total), it is possible to calculate the amount of energy needed. The kWh must first be estimated based on the SAE (Standard Aeration Efficiency) and then based on the OTR (Real Oxygen Transfer Rate), as follows: kWh SAE = g O2 total*/*670 where, 670 is the average standard aeration efficiency of diffuse air in grams of oxygen per kWh, commonly used in BFT systems. Then the OTR can be calculated: kWh OTR = kWh SAE*/*0*.*6 where, 0.6 is a correction factor considering a situation close to the experiment, where the amount of oxygen in the water is at least 4 mg ∙ L^-1^ and the water temperature is around 26 to 28 ºC.

### Statistical analysis

The efficiency of masculinization was assessed by the chi-square test with Bonferroni correction, as the response variable is categorical (male, female, intersex, undefined). Regression models were fitted to describe the effects of stocking densities on water quality, growth performance and economic variables (p < .05). The goodness of fit of regression models was evaluated by both residual analysis and determination coefficient (R^2^). The validity of the models was assessed by the analysis of homoscedasticity (Bartlett’s test) and normality (Shapiro-Wilk test) of residuals.

To compare body size uniformity among the stocking densities at the end of the first phase (28 days of hormone treatment), the individual measurements of final body weight of the 30 individuals per tank (120 individuals per treatment) were used and the homogeneity of variances were tested by Bartlett and F-test to compare the five variances.

All statistical analyses were performed using RStudio software version 2023.06.2+561 (R Core Team, 2023) and InfoStat program (Di Rienzo et al., 2015).

## Results

### Water quality

Water quality parameters remained within the recommended range for Nile tilapia (Table 1), except for TSS, which showed values above 1,000 mg ∙ L^-1^ in most treatments (Avnimelech, 2009), possibly due to zero water exchange during the experimental period, with water replenished only for evaporation losses. The exception of SS, pH, TOC and its differences with the initial amount of carbon, all other water quality variables were not affected by the increasing of the stocking density. The pH and SS showed a negative and positive linear pattern, respectively, as stocking density increased. Meanwhile, total organic carbon and its difference from the initial carbon followed quadratic patterns likely the results of total feed intake per treatment (Table 2). They decreased and above 3.74 larvae ∙ L^-1^ (minimum point) they increased following the increase in stocking density.

**Table 1:** Water quality variables (mean ± standard deviation) and initial and final values (in parentheses) for Nile tilapia larvae reared in biofloc system for 28 days of masculinization under different stocking densities.

**Variables**	**Stocking density (larvae ∙ L^-1^)**					***p*-value**		**Regression models; R^2^** ^*^
	**1.5**	**3**	**4.5**	**6**	**7.5**	**d**	**d^2^**	
Temperature (°C)	29.14±0.70 (28.93-29.5)	28.42±0.45 (28.8-29.13)	28.76±0.41 (28.7-28.85)	29.06±0.50 (28.9-29.05)	28.54±0.40 (28.43-28.55)	.6320	.7180	Y= 28.96
Dissolved oxygen (mg ∙ L^-1^)	7.93±0.34 (7.11-7.92)	7.78±0.22 (7.03-8.13)	7.93±0.20 (7.64-8.19)	7.91±0.25 (7.27-8.24)	7.75±0.24 (7.17-8.09)	.7900	.6960	Y= 7.93
pH	8.19±0.03 (7.66-8.53)	8.26±0.02 (7.68-8.53)	8.19±0.08 (7.68-8.53)	8.12±0.06 (7.68-8.53)	8.10±0.12 (7.64-8.54)	.0162	.239	Y= 8.26 - 0.02d; R^2^= .24
Salinity (g ∙ L^-1^)	0.91±0.06 (0.27-1.33)	0.92±0.03 (0.27-1.23)	0.90±0.04 (0.27-1.20)	0.94±0.06 (0.27-1.28)	0.95±0.02 (0.26-1.35)	.5850	.4230	Y= 0.90
Alkalinity (mg of CaCO_3_ ∙ L^-1^)	64.48±4.31 (35-82.5)	69.27±4.21 (40-82.5)	66.04±7.46 (45-82.5)	65.25±8.46 (47.5-82.5)	60.63±2.92 (35-82.5)	.2200	.1380	Y= 68.65
TAN (mg ∙ L^-1^)	0.01±0.01 (0.01-0.03)	0.02±0.02 (0.01-0.03)	0.02±0.01 (0.01-0.01)	0.01±0.01 (0.01-0.05)	0.11±0.17 (0.01-0.08)	.3440	.2120	Y= 0.02
NH_3_ (mg ∙ L^-1^)	0.0024±0.0025 (0.0017-0.0021)	0.0034±0.0052 (0.0009-0.002)	0.0031±0.0015 (0.0015-0.0021)	0.0008±0.0003 (0.001-0.0021)	0.0044±0.0054 (0.0014-0.0021)	.6390	.6810	Y= 0.0024
Nitrite (mg ∙ L^-1^)	0.36±0.17 (0.08-0.17)	0.35±0.10 (0.08-0.23)	0.36±0.15 (0.08-0.20)	0.35±0.13 (0.08-0.11)	0.36±0.09 (0.08-0.29)	.8458	.8469	Y= 0.36
Nitrate (mg ∙ L^-1^)	251.69±56.30 (16.8-486.57)	192.89±15.24 (16.8-368.99)	222.24±47.89 (16.8-427.68)	248.02±86.27 (16.8-479.24)	282.35±69.80 (16.8-547.9)	.1778	.1105	Y= 204.50
Settleable solids (ml ∙ L^-1^)	11.53±3.93 (8-20.5)	10.72±1.85 (8-16)	13.05±5.51 (8-30.25)	21.6±11.91 (8-57)	27.45±10.44 (8-56.25)	.0022	.1739	Y= 4.05 + 2.85d; R^2^= .38
Total suspended solids (mg ∙ L^-1^)	1071.53±104.61 (636.8-1506.25)	1022.15±106.82 (636.8-1407.5)	998.40±224.94 (636.8-1360)	1021.53±212.86 (636.8-1406.25)	1259.03±226.73 (636.8-1881.25)	.1298	.0761	Y= 962.21
Phosphate (mg ∙ L^-1^)	19.52±1.62 (4.52-34.53)	18.99±1.02 (3.45-34.53)	18.30±0.37 (2.08-34.53)	18.35±0.63 (2.17-34.53)	18.56±0.68 (2.59-34.53)	.1240	0.2120	Y= 19.51
Initial total organic carbon (mg ∙ L^-1^)	12.34	12.34	12.34	12.34	12.34	-	-	
Total organic carbon (mg ∙ L^-1^)	32.44±4.26	21.60±2.18	25.16±1.03	27.40±1.93	58.85±2.69	< .0001	< .0001	Y= 56.23 – 19.23d + 2.57d^2^; R^2^= .90
Total organic carbon difference (final – initial) (mg ∙ L^-1^)	20.10±4.26	9.26±2.18	12.82±1.03	15.06±1.93	46.51±2.69	< .0001	< .0001	Y= 43.89 – 19.23d + 2.57d^2^; R^2^= .90

* All regression coefficients presented were significant (*p* < .05); d = stocking density (larvae ∙ L^-1^). Reference values: T (°C): 27-32 (El-Sayed, 2006); OD: > 4 mg ∙ L^-1^; pH: 6-9 (Wedemeyer, 1996); Salinity:1-8 g ∙ L^-1^ (Alvarenga et al., 2018); Alkalinity: > 50 mg of CaCO_3_ ∙ L^-1^ (Avnimelech, 2009); ), Total ammonia nitrogen (TAN):< 1 mg ∙ L^-1^ (El-Sayed, 2019); NH_3_: < 0.1 mg ∙ L^-1^ (El-Sayed, 2019); Nitrate<500 mg ∙ L^-1^ (Monsees et al., 2017); Settleable solids (ml ∙ L^-1^): <50 ml∙ L^-1^ ; Total suspended solids: <1000 mg ∙ L^-1^ (Avnimelech, 2009); Nitrite NO^2-^: < 8 (El-Sayed, 2019).

**Table 2:** Growth performance and survival variables (mean values ± standard deviation) for Nile tilapia larvae reared in biofloc system for 28 days of masculinization under different stocking densities.

**Variables**	**Stocking density (larvae ∙ L^-1^)**					***p*-value**		**Regression models; R^2^** ^*^
	**1.5**	**3**	**4.5**	**6**	**7.5**	**d**	**d^2^**		
Total feed intake (g)	104±2.58	89.25±2.75	96.50±3.42	113.50±1.29	123.75±1.26	.0002	< .0001	Y= 116.15 - 12.82d + 1.906d^2^; R^2^ = .85
Individual feed intake (g)	1.69±0.08	0.78±0.05	0.63±0.02	0.67±0.14	0.58±0.03	< .0001	< .0001	Y= 2.49 - 0.68d + 0.06d^2^; R^2^ = .87
Final weight (g)	0.51±0.07	0.26±0.04	0.2±0.07	0.19±0.03	0.16±0.03	< .0001	.0004	Y= 0.73 - 0.19d + 0.02d^2^; R^2^ = .84
Final productivity (kg ∙ m^-3^)	0.63±0.11	0.59±0.06	0.62±0.21	0.65±0.12	0.69±0.10	.5780	.4490	Y= 0.58
Weight gain (mg ∙ day^-1^)	17.72±2.48	8.9±1.45	6.91±2.60	6.34±1.19	5.4±0.94	< .0001	.0004	Y= 25.8 - 6.72d + 0.54d^2^; R^2^ = .84
Final biomass (g)	31.31±5.67	29.32±2.91	30.85±10.16	32.4±5.85	34.56±5.09	.5990	.4670	Y= 28.82
Specific growth rate (% ∙ day^-1^)	13.8±0.85	11.36±0.75	10.26±1.11	10.3±0.48	9.81±0.74	.0003	.0040	Y= 16.33 - 2.04d + 0.16d^2^; R^2^ = .78
Final length (cm)	2.92±0.10	2.34±0.14	2.21±0.26	2.19±0.08	2.14±0.05	< .0001	.0009	Y= 3.45 - 0.45d + 0.04d^2^; R^2^ = .76
Survival (%)	82±3.85	75.67±4.37	67.67±3.29	58.1±10.05	57.2±2.38	< .0001	.3666	Y= 88.25 - 4.47d; R^2^ = .77

* All regression coefficients presented were significant (*p* < .05); d = stocking density (larvae ∙ L^-1^).

### Growth performance and sexual proportion

**Figure 1:**
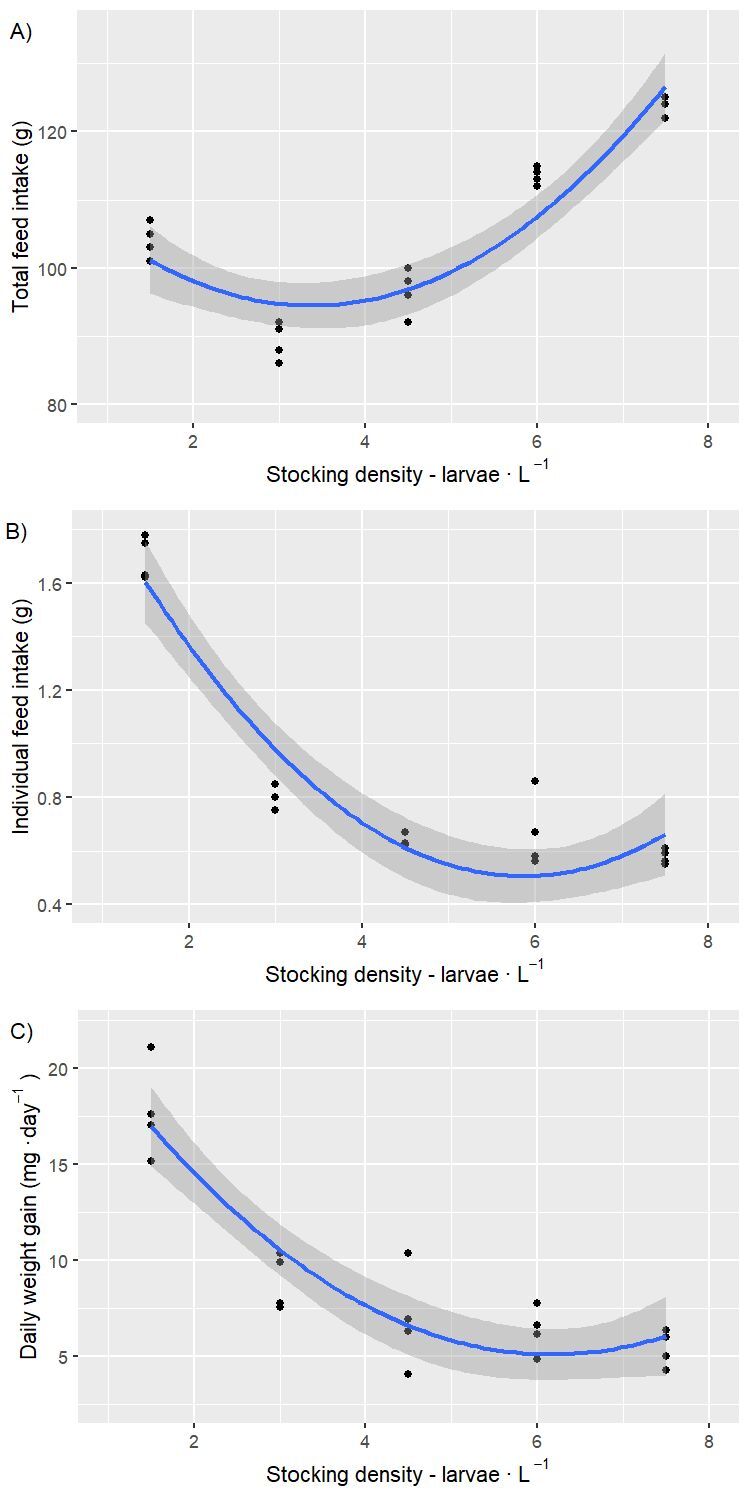
A) Total feed intake (g) as a function of stocking densities of Nile tilapia larvae reared in biofloc system during masculinization period; B) Individual feed intake (g) as a function of stocking densities of Nile tilapia larvae reared in biofloc system during masculinization period; C) Daily weight gain (mg ∙ day^-1^) as a function of stocking densities of Nile tilapia larvae reared in biofloc system during masculinization period.

In the evaluation of growth performance at 28 days of experiment (Table 2), total feed intake (Figure 1A), individual feed intake (Figure 1B), weight gain (Figure 1C), final weight, specific growth rate, and final length were fitted by a positive quadratic polynomial model as a function of the increase of the stocking density. The minimum points derived from the quadratic regression were close to 6 larvae ∙ L^-1^, with exception for total feed intake (3.37 larvae ∙ L^-1^) and final weight (4.75 larvae ∙ L^-1^). According to the Bartlett and F-test, the uniformity of the final body weights of fingerling at 28 days of experiment was not different among the stocking densities.

The highest survival rate for *O. niloticus* larvae was obtained at a stocking density of 1.5 larvae ∙ L^-1^, once this variable linearly decreased as stocking density increased in the first phase (Table 2). And in the second phase, the highest survivals were also for this treatment, even all stocking densities being decreased to 1.5 larvae ∙ L^-1^ (Table 3).

**Table 3:** Cost evaluation (mean values ± standard deviation) of the masculinization of Nile tilapia in biofloc system under different stocking densities. Estimated costs (US$) to produce a thousand fingerlings of 28 days of age (at the end of the masculinization period) and of 1g of body weight.

**Variables**	**Stocking density (larvae ∙ L^-1^)**					***p*-value**		**Regression models, R^2^** ^*^
	**1.5**	**3**	**4.5**	**6**	**7.5**	**d**	**d^2^**	
**Costs for a thousand fingerlings of 28 days of age**								
Cost of procurement of larvae	7.33±0.35	7.95±0.45	8.89±0.43	10.60±2.07	10.49±0.44	< .0001	.725	Y = 6.36 + 0.6d; R^2^ = .63
Feed cost	2.71±0.13	1.26±0.08	1.02±0.04	1.07±0.22	0.92±0.04	< .0001	< .0001	Y = 3.98 - 1.08d + 0.09d^2^; R^2^ = .87
Energy for aeration cost	0.63±0.03	0.3±0.02	0.24±0.01	0.25±0.05	0.22±0.01	< .0001	< .0001	Y = 0.93 – 0.25d + 0.02d^2^; R^2^ = .88
Hormone cost	0.65±0.03	0.31±0.02	0.25±0.01	0.26±0.05	0.22±0.01	< .0001	< .0001	Y = 0.95 – 0.26d + 0.02d^2^; R^2^ = .88
Alcohol cost	1.02±0.05	0.47±0.03	0.38±0.01	0.40±0.08	0.35±0.01	< .0001	< .0001	Y = 1.49 – 0.41d + 0.03d^2^; R^2^ = .87
Limestone cost	0.13±0.01	0.06±0.03	0.05±0.01	0.05±0.01	0.04±0.00	< .0001	.0002	Y = 0.19 – 0.05d + 0.004d^2^; R^2^ = .81
Salt cost	0.08±0.01	0.05±0.01	0.04±0.01	0.03±0.01	0.02±0.00	< .0001	.0003	Y = 0.11 – 0.03d + 0.002d^2^; R^2^ = .89
Sugar cost	0.0±0.00	0.0±0.00	0.0±0.00	0.0±0.00	0.0±0.01	.3607	.2286	Y= 0.0020
Total variable cost	12.55±0.58	10.38±0.57	10.85±0.48	12.65±2.48	12.27±0.52	.471	.0709	Y= 11.23
Survival from the 28^th^ day to 1g of body weight (%)	80.53±12.11	62.67±6.62	54.0±10.97	58.67±8.33	50.22±13.42	.0026	.1064	Y = 80.99 – 4.48d; R^2^ = .41
Days to achieve 1 g	8.75±4.86	11.25±2.99	13.0±3.65	20.33±9.29	20.33±2.31	.0010	.859	Y = 5.05 + 2.13d; R^2^ = .47
**Costs for a thousand fingerlings of 1g of body weight**								
Cost of procurement of 28-day fingerlings	15.88±29.65	16.76±2.55	20.65±3.70	19.76±2.55	25.85±8.79	.0055	.5773	Y= 12.99 + 1.52d; R^2^ = .35
Feed cost (from 28^th^ day to 1g)	1.56±0.81	1.69±0.42	1.87±0.51	2.92±1.44	2.85±0.38	.0087	.666	Y = 1.05 + 0.25d; R^2^ = .32
Energy for aeration cost (from 28^th^ day to 1g)	0.36±0.19	0.39±0.10	0.44±0.12	0.68±0.33	0.66±0.09	.0087	.666	Y = 0.24 + 0.06d; R^2^ = .32
Total variable cost	17.81±3.24	18.85±2.61	22.95±3.70	23.36±3.03	29.36±8.54	.0014	.5154	Y = 14.28 + 1.83d; R^2^ = .45

* All regression coefficients presented were significant (*p* < .05); d = stocking density (larvae ∙ L^-1^).

As the stocking density increases, there was also an extension of the period required for the fingerlings to reach 1g (Table 3) following the final body weight results achieved at the end of the first phase.

**Figure 2:**
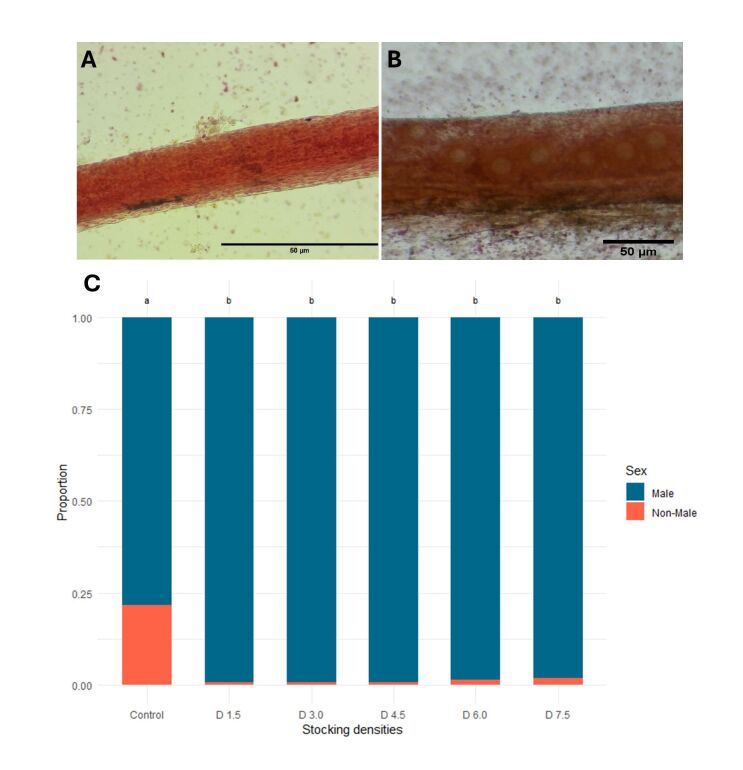
Results of sexing analysis of Nile tilapia under different stocking densities (from 1.5 to 7.5 larvae ∙ L^-1^ represented by D 1.5, D 3.0, D 4.5, D 6.0 and D 7.5) during the masculinization phase with 17α-methyltestosterone in biofloc system. A) Microscopic image of a male gonad subjected to the aceto-carmine technique from an animal in the control group of Nile tilapia produced using biofloc system; B) Microscopic image of female gonad subjected to the aceto-carmine technique from an animal the control group of Nile tilapia in BFT; C) Proportion of male and non-male fingerlings of Nile tilapia submitted to different stocking densities in the masculinization period in biofloc system. The proportions of non-male obtained for control, 1.5, 3.0, 4.5, 6.0 and 7.5 larvae ∙ L^-1^ were 21.74, 0.5, 0.56, 0.52, 1.09 and 2.96%, respectively. As there were no differences between the proportions of males in the treatments, the average result was 98.87%.

The male proportions were similar among stocking densities (average = 98.87%), but all treatments differed from the control (Figure 2). The proportion of non-males (females, intersex, and undetermined) in the control treatment, without the presence of hormone in the diet, was 21.74%, while in treatments with different stocking densities, the highest observed value was 2.96% (average = 1.13%).

### Method validation and MT residues

Chromatograms of the blank samples were compared with those of the fortified samples. The absence of signals that could be attributed to matrix interferents, considering their retention times and signal-to-noise ratio, confirmed the effectiveness of the method's selectivity (Ribani et al., 2004). The inter- and intra-day variability for different levels of MT concentration in samples was used to evaluate precision and accuracy (Table 4). The precision values, expressed as relative standard deviation (RSD), ranged from 6.282% to 8.813%, within the acceptable limit of 20% as stipulated by INMETRO (2020). The accuracy, measured by recovery, ranged from 70.79% to 99.88%, within the acceptable range of 70 to 120% (Ribani et al., 2004; INMETRO, 2020). These results demonstrate that the developed method is highly sensitive, precise, and accurate in analyzing MT in biofloc water.

**Table 4:** Intra-day and inter-day accuracy (mean ± standard deviation) and precision of methyltestosterone measurement using high performance liquid chromatography (HPLC) in biofloc water at three different levels.

Methyltestosterone	Intra-day (*n*= 6)		Inter-day (*n*= 6)	
Spiked concentration (µg ⋅ L^−1^)	Measured concentration (µg ⋅ L^−1^)	Precision (RSD, %)	Measured concentration (µg ⋅ L^−1^)	Precision (RSD, %)
50	35.665 ± 2.967	8.321	35.393 ± 3.119	8.813
100	101.065 ± 7.595	7.515	99.880± 6.986	6.995
200	152.932± 11.964	7.823	153.630± 9.651	6.282

The analytical calibration curve of MT exhibited satisfactory linearity, with a linear regression equation of Y= 1267.3 + 112.82×C, where C is the concentration of MT in µg ⋅ L^−1^, resulting in a correlation coefficient (r) of 0.999 and a determination coefficient (R^2^) 0.998. The curve construction was performed with six levels of MT concentration (25, 50, 100, 200, 400, 600, 1000, 1500, 2000, 2500 µg ⋅ L^−1^), as recommended by regulatory bodies such as the International Conference on Harmonization (ICH), National Health Surveillance Agency (ANVISA, 2003), and the Pesticide Residue Analysts Group (GPRA) (ICH, 1994; ANVISA, 2003). The limit of quantification (LOQ) of MT in biofloc water was determined to be 25 µg ⋅ L^−1^ based on S/N = 10, while the limit of detection (LOD) was established at 10 µg ⋅ L^−1^, corresponding to S/N = 3. These results confirm the suitability of the method for quantifying MT in biofloc water.

In the analysis of matrix effect, no statistically significant differences were found between the analytical curves of solvent standards and the curves in the matrix. Therefore, it was chosen to validate the method using curves constructed in the solvent.

Considering individual feed intake (a result of total amount feed intake and survival), the amount of feed to produce a thousand masculinized fingerlings of 28 days of age would be 1.693, .788, .634, .669 and .577 g of feed enriched of MT for stocking densities of 1.5, 3.0, 4.5, 6.0, and 7.5 larvae ∙ L^-1^, respectively. Consequently, the amount of MT to produce a thousand masculinized fingerlings of 28 days of age would be 101.61, 47.28, 38.07, 40.15, and 34.62 mg of MT for stocking densities of 1.5, 3.0, 4.5, 6.0, and 7.5 larvae ∙ L^-1^, respectively. Since the estimate of hormone input in the system is proportional to individual consumption (the concentration of MT is the same for all levels), the minimum point of MT input is also close to 6 larvae ∙ L^-1^, even considering the survival to produce a fingerling of 1g of body weight (Table 3).

The amount of MT that entered the system throughout the entire experiment were 124.8, 107.1, 115.8, 136.2 and 148.5 µg of MT ∙ L^-1^ for stocking densities of 1.5, 3.0, 4.5, 6.0, and 7.5 larvae ∙ L^-1^, respectively. None of the biofloc water samples showed detectable residues of MT, even though the lower MT input was over 10x times the limit of its detection (the minimum value for detection).

### Variable cost evaluation

Positive quadratic effects of stocking density on inputs (Table 3) were observed but did not affect total variable costs for a thousand masculinized fingerlings up to 28-day (mean value = US$ 11.23). The minimum points derived from the models were also close to 6 larvae ∙ L^-1^, except for the cost of procurement of larvae that followed a positive linear regression, and the highest cost was obtained at 7.5 larvae ∙ L^-1^. In the second phase, when the fingerlings were maintained at the same stocking density (1.5 larvae ∙ L^-1^), costs for feed, energy and 28-day fingerling procurement increased following the stocking density, which led to a linear increase in total variable costs to produce a thousand 1g fingerlings for higher stocking densities.

## Discussion

Despite environmental concerns associated with hormone use, masculinization protocol using MT in feed is widely adopted due to its high efficiency, simplicity, and low cost (Baroiller et al., 2009; Baroiller and D’Cotta, 2018). Considering the biofloc system, which involves little to no water discharge into the environment, there is potential to reduce the environmental issues relating to hormone use. This highlights the viability of BFT as a more sustainable alternative to addressing the challenges associated with early production of tilapia, providing also environmental benefits. Our results showed that it is possible to produce batches of masculinized Nile tilapia fingerlings (over 97.4% of males) based on a protocol of 60 mg of MT ∙ kg^-1^ of feed with zero water exchange, up to 7.5 larvae ∙ L^-1^ of stocking density on a BFT system, and with undetectable MT residues at the end of the masculinization period. However, survival and economic analysis suggest a lower stocking density of 1.5 larvae ∙ L^-1^, to achieve the best results in growth performance and lower variable costs for fingerlings with size for sale.

### Water quality

In this study, no water exchange was performed, only water lost through evaporation was replenished, thus characterizing a typical zero water exchange system. The ideal pH for an efficient biofloc system is generally between 7.5 and 8.0, a crucial value due to its impact on the activity of nitrifying bacteria (Abakari et al., 2021). The values found in the study were close to the recommended range, but they showed a small decrease with increasing stocking density, possibly due to carbonic gas production during respiration by an increase of the number of fish.

The greater the amount of feed provided and stocking density, the more nutrients (nitrogen and carbon) are added in the system and could result in negative effects on larvae performance and survival in BFT (Manduca et al., 2020 and 2021). The results obtained for water quality are consistent with previous studies that observed an increase in SS at higher stocking densities. This is attributed to the increased amount of feed, a source of organic carbon, and animal feces (Haridas et al., 2017; Liu et al., 2018; Zaki et al., 2020; Aliabad et al., 2022), that increased substrate available for heterotrophic bacterial growth, resulting in these higher concentrations of settleable solids (Perez-Fuentes et al., 2016).

Stocking density is considered an important factor affecting microbial communities in BFT systems (Khanjani and Sharifinia, 2022). Carbon source is inevitable at higher stocking density to maintain the C:N ratio (Deng et al., 2019). Usually, the C:N ratio is regulated by the external supply of carbohydrates according to the nitrogen and carbon content in the provided feed (Avnimelech, 1999). Considering that, in this study, the main source of carbon input was through exogenous feeding (practically additional carbon source was not used), this would possibly explain the initial decrease and followed increase of total final carbon as the stocking density rose, as observed in the total feed consumption per treatment (Table 2 and Figure 1A). In fact, only once during the experimental period, in a tank with a density of 7.5 larvae ∙ L^-1^, addition of cane sugar was necessary to stimulate the formation of heterotrophic bacteria and bioflocs, due to an ammonia spike. In the other treatments, TAN correction was not required. Thus, the amount of organic carbon added to each experimental tank can be estimated, considering that there was no water exchange during the entire masculinization period, using the average amount of feed offered (104,000; 89,250; 96,500; 113,500; 123,750 mg of feed for each treatment), the proportion of dry matter in the initial feed (88%), the average proportion of organic carbon in the dry matter of the initial feed (41.36%; Chatvijitkul et al., 2017), and the average proportion of organic carbon from the initial feed that is not retained in the fish (81.7%; Chen et al., 2015): mg of feed ∙ 88% ∙ 41.36% ∙ 81.7% ∙ 28 days^-1^ ∙ 50 L^-1^ = 22.09; 18.96; 20.50; 24.11; 26.28 mg ∙ L^-1^, respectively for stocking densities of 1.5; 3; 4.5; 6; and 7.5 larvae ∙ L^-1^.

When considering that between 18.96 to 26.28 mg of organic carbon were added to the tanks mainly through feed, the difference of over 45 mg ∙ L^-1^ between initial and final measurements of organic carbon (Table 1) at the highest density can largely be attributed to carbon retention carried out by autotrophic organisms. This result indicates that BFT, even over short periods, can contribute to carbon retention, as demonstrated by Chen et al. (2015) and Zhang et al. (2020).

### Growth performance and survival

Even when feed was supplied at a rate of ≥15% of the biomass, the increase in stocking density significantly affected fry growth performance — an expected result, as higher densities generally intensify competition and stress. Consistent with these results, other studies show that regardless of the growth stage, increased stocking density tends to reduce the growth and survival of fish in different systems (El-Sayed, 2002; Ferdous et al., 2014; Tachibana et al., 2009; Manduca et al., 2020; Manduca et al., 2021). One of the factors that may result in reduced mass gain is stress at high stocking densities, which, depending on the degree and intensity of growth, can permanently impair future growth performance (Mélard et al., 1997). Frequently, stress resulting from crowding, social interactions, and competition for food can reallocate metabolic energy intended for growth to restore homeostasis (Martínez-Porchas et al., 2009). In this scenario, increasing fish stocking density typically prolongs the time required for tilapia to reach selling weight (Garcia et al., 2013; Manduca et al., 2021), in agreement to our results.

In the present study, the average final body weight at 28 days of culture was 0.51, 0.26, 0.2, 0.19, and 0.16 g, respectively, from the lowest to the highest stocking density. This variation was also observed by Phelps et al. (1995), who stocked larvae at densities of 75, 200, and 260 larvae ⋅ m^-2^ in ponds and treated them with a diet containing 60 mg ∙ kg^-1^ of methyltestosterone for 28 days, resulting in juveniles of different sizes, weighing 2.77 g, 0.84 g, and 0.86 g, respectively. The same large variation was also obtained in our study for specific growth rate, where the highest specific growth rate (13.8% ∙ day^-1^) was recorded at the lowest stocking density, while the lowest value (9.81% ∙ day^-1^) was observed at the highest stocking density. This aligns with the findings of Alam et al. (2011) and Ferdous et al. (2014), as the survival findings are consistent with Chakraborty and Banerjee (2010) and Vera Cruz and Mair (1994), which reported higher survival rates at lower stocking densities. These studies also indicated that fish survival decreases as stocking density increases probably due to stress, poor management, and intense competition for food and space, and according to our results, negative effects of the highest stocking densities in the masculinization phase remained in the second one, even the stocking densities being decreased to the lowest one. In traditional systems, such as hapas at earthen ponds or RAS, stocking densities similar to those tested have likewise resulted in comparable mortality rates, which vary depending on the strain and management practices. In practice, producers generally stock up to 5 larvae · L^-1^, avoiding higher densities because of the associated risk of increased mortality (Popma and Green, 1990; Sanches and Hayashi, 1999; Vera Cruz and Mair, 1994).

### Masculinization proportion e MT residues in the biofloc water

The results of this study are similar to those described by Tachibana et al. (2009), that also did not find significant difference in tilapia masculinization (>96%) at different stocking densities (1, 3, 5, and 7 larvae ∙ L^-1^) in a recirculating aquaculture system. Our result of masculinization (98.87% of males) was higher than those obtained by Tachibana et al. (2009) and Costa e Silva et al. (2022). Nile tilapia exhibits genetic sex determination (XX–XY system) with environmental influences. Although sex is genetically determined, it can be altered by factors such as temperature and hormones during the labile period of sexual differentiation, which occurs approximately between 5 and 21 days post-hatching (Baroiller and D’Cotta, 2018). Therefore, the average temperature of 28°C used in the present study may have contributed to the higher proportion of males observed in the control group (>50%), even in the absence of hormones in the feed, suggesting that this strain could be more sensitive to temperature. Similar results were reported by Ramirez et al. (2024) and are consistent with the findings of Bezerra et al. (2025), who observed high thermosensitivity in Nile tilapia strain used in present work. Further studies involving different strains of Nile tilapia are recommended to more accurately assess the effects of the culture system, masculinization protocol, and genetic background on the proportion of males.

The absence of MT 12 hours after the last feeding with hormone in the BFT in this study is an important step in terms of economic and environmental aspects. These results suggest that the biofloc system may contribute to degrade methyltestosterone from water and consequently reducing effluents with hormone residues in the environment. Possibly, the microbial community present in the bioflocs is responsible for the biodegradation of androgens (Homklin et al., 2009; Homklin et al., 2012). In the present study, microbiological analysis of the biofloc was not performed, however, recent studies have identified that the biofloc system presents different genera of MT-degrading bacteria, including *Nocardioidaceae* and *Rhodococcus* (Rajeev et al., 2023). Another possible explanation for the absence of hormone detection in the water is that MT concentrations were below the analytical detection limit of 10 µg L^−1^ used in this study, or that the fish consumed the MT-enriched feed before the hormone degraded or dissipated in the water due to feeding competition, which could also have contributed to the high masculinization levels observed. Since no additional filtration system was employed and the collected water samples contained both suspended bioflocs and sediment, it is likely that, had uneaten feed remained in the system, higher hormone concentrations would have been detected. Further studies are needed to confirm whether the hormone persists at concentrations below the 10 µg L^−1^ detection limit.

### Variable cost evaluation

The reduction of the larvae weight gain and the higher mortality up to the end of the masculinization period as the stocking density increased lead to a reduction of the most variable costs to produce a thousand of 28-day fingerlings. However, it is important to draw attention that the lower survival promoted an increase of the cost of procurement of initial larvae, so there was no difference in total variable cost.

It is evident that the impact of the stocking density in the first phase of larviculture continued to negatively affect the survival of the animals at the second one. Again, the lower survival in higher stocking densities leads to an increase in the cost of procurement of 28-day fingerlings for the second phase. Besides, the lower final weight at the end of the masculinization period due to higher stocking densities promoted an increase of feed and energy for aeration costs in this phase too, which also promoted an increase of the total variable cost to produce a thousand 1g fingerlings.

In addition to the direct economic factor, stocking density significantly influences the final size and uniformity of fingerlings (Dambo and Rana, 1993). When it comes to large-scale production of juveniles for commercialization, both the desired size and uniformity are of paramount importance, as these aspects directly reflect the quality and therefore the value of the fingerling in the market, provided satisfactory masculinization rates are maintained (Sanches and Hayashi, 1999). In the present study, although the stocking densities affected the body weight on the 28^th^ day after yolk sac absorption, the uniformity of fingerling was not different among the stocking densities.

The increase of stocking density can directly impact on the sales of fingerlings, especially when the fingerlings fail to reach the average commercial weight of 1g for Nile tilapia. Upon analyzing the time required to reach this weight, it becomes evident in the present study that increasing stocking density results in a longer production period. Consequently, this leads to additional costs as it requires more time occupying tanks, labor, and feed. Furthermore, considering that animal survival, beyond a density of 1.5 larvae ∙ L^-1^, was below 80%, there is a need to maintain a more robust broodstock/reproduction unit to ensure larval production.

However, it is important to emphasize that this study was conducted on a commercial test scale, which still differs from a full commercial production scale. Variable production costs that are immediately and proportionally affected by stocking density—such as feed, electricity, and larval acquisition—were taken into account. Even on this scale, these costs are comparable to those observed in large-scale operations and represent a significant portion of the total production cost.

Moreover, the results are based on a system with zero water exchange, reflecting the use of this important natural resource in a way that promotes greater system sustainability through reduced consumption and controlled disposal of organic waste, which may still contain hormonal residues.

Nevertheless, more detailed economic feasibility studies are essential, particularly at the commercial scale, incorporating factors such as labor, fixed costs, and investment, which directly depend on the number and size of tanks as well as their depreciation. These elements are crucial for supporting strategic decisions, since lower densities—although favoring survival—do not optimize the use of available space, requiring a larger number of tanks to achieve the desired fry production.

## Conclusion

Increasing the stocking density from 1.5 to 7.5 larvae ∙ L^−1^ in the masculinization of Nile tilapia using 60 mg of 17α-methyltestosterone ⋅ Kg^−1^ of feed, in zero water exchange BFT, negatively affected its growth and survival. Therefore, stocking density of 1.5 larvae ∙ L^−1^ resulted in a lower total variable cost to produce a thousand fingerlings of 1g of body weight and proved effectiveness in achieving masculinization rate above 99%. Since no hormone residues were detected, even when using strict quantification and detection limits, this study suggests that the biofloc system may contribute to in degrading methyltestosterone from water. More detailed economic feasibility studies are essential to support decisions regarding production intensification, particularly concerning the compensation of larval losses and the economic sustainability of the hatchery system.
